# Direct dilation-free stent delivery using a novel 0.035-inch guidewire in endoscopic ultrasound-guided biliary drainage: the new ‘Flush surface’ method

**DOI:** 10.1055/a-2868-3185

**Published:** 2026-06-15

**Authors:** Kosuke Takahashi, Eisuke Ozawa, Mizuki Kitagawa, Masashi Shibata, Hisamitsu Miyaaki

**Affiliations:** 1 Gastroenterology and Hepatology 200674 Nagasaki University Graduate School of Biomedical SciencesNagasakiJapan

Stent delivery following guidewire placement is the most technically demanding step of endoscopic ultrasound-guided biliary drainage (EUS-BD 1 2 3 ). Although various devices can dilate endosonography-created routes, excessive tract dilation increases the risk of bile leakage 4 5 , making stable stent delivery without additional tract dilation desirable.

**Fig. 1:**
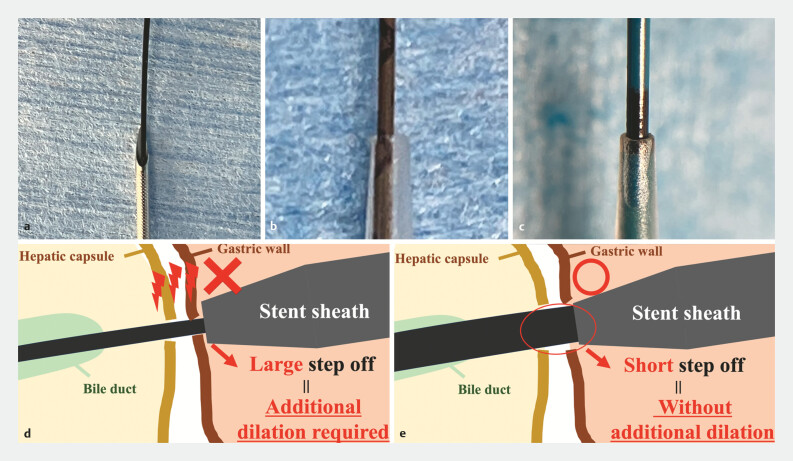
Comparison of outer diameters of different guidewires and the step-off configuration. **a** Comparison of the novel 0.035-inch guidewire (0.65 mm) and the conventional 0.035-inch guidewire (approximately 0.8 mm), demonstrating compatibility with a 19-gauge needle (inner diameter of approximately 0.7 mm). **b, c** Images comparing a conventional 0.025-inch guidewire (left) and the novel 0.035-inch guidewire (right), highlighting the reduced step-off between the novel guidewire and the inner sheath of the stent delivery system. **d, e** Schematic illustrations comparing the conventional 0.025-inch guidewire (left) and novel 0.035-inch guidewire (right), demonstrating how the minimized step-off with the novel guidewire facilitates direct stent delivery without any additional tract dilation.

Conventional 0.035-inch guidewires generally exceed 0.75 mm in outer diameter, limiting compatibility with 19-gauge needles (inner diameter ~0.7 mm); this novel 0.035-inch guidewire has a reduced outer diameter (0.65 mm), enabling insertion through a 19-gauge needle while preserving high shaft stiffness ( Fig. 1 **a** ). Compared with 0.025-inch guidewires ( Fig. 1 **b** ), the larger outer diameter minimizes step-off between the guidewire and the stent delivery system, potentially improving coaxial alignment and pushability during stent advancement ( Fig. 1 **c** ). Here, we describe the “flush surface method,” compatible with a 19-gauge needle which enables smooth stent delivery without additional tract dilation ( Fig. 1 **d, e** ; Video 1 ).

The flush surface method in EUS-guided biliary drainage. EUS, endoscopic ultrasound.Video 1

**Fig. 2:**
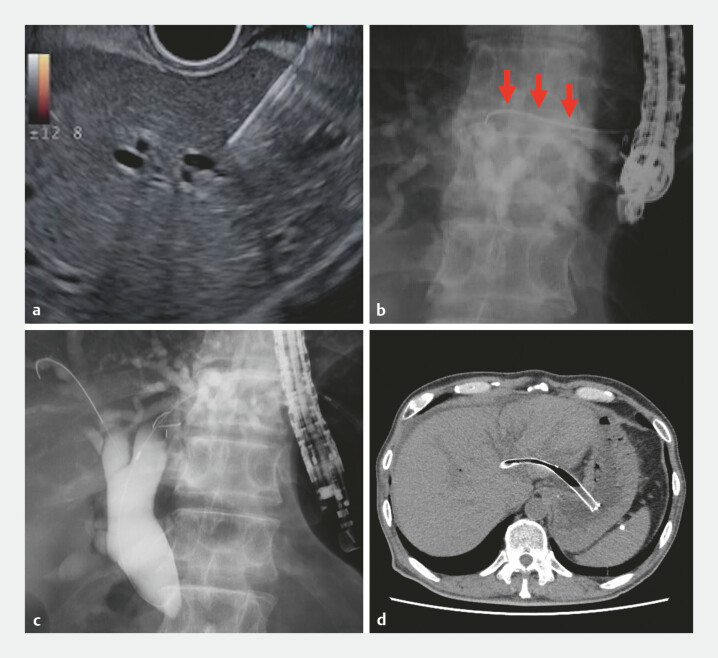
EUS-guided hepaticogastrostomy using the novel guidewire in the first patient. **a** Contrast injection following 19-gauge puncture of the intrahepatic bile duct. **b** Direct insertion of the novel 0.035-inch guidewire through the 19-gauge needle. **c** Fluoroscopic image showing smooth delivery of a self-expandable metal stent without tract dilation. **d** CT scan confirming appropriate stent placement.

A 60-year-old woman with obstructive jaundice caused by pancreatic cancer was referred for EUS-guided hepaticogastrostomy (EUS-HGS). A 19-gauge needle (EZ Shot 3 Plus; Olympus, Tokyo, Japan) punctured the B2 intrahepatic bile duct. After contrast injection ( Fig. 2 **a** ), the novel 0.035-inch guidewire (CAPELLA 0.035; Japan Lifeline, Tokyo, Japan) was directly inserted through the needle, advancing into the common bile duct ( Fig. 2 **b** ). Because of its stiff shaft and reduced step-off, the self-expandable metal stent delivery system advanced smoothly without a dedicated tract dilation device ( Fig. 2 **c** ). Stent deployment was successful ( Fig. 2 **d** ), with no adverse events.

**Fig. 3:**
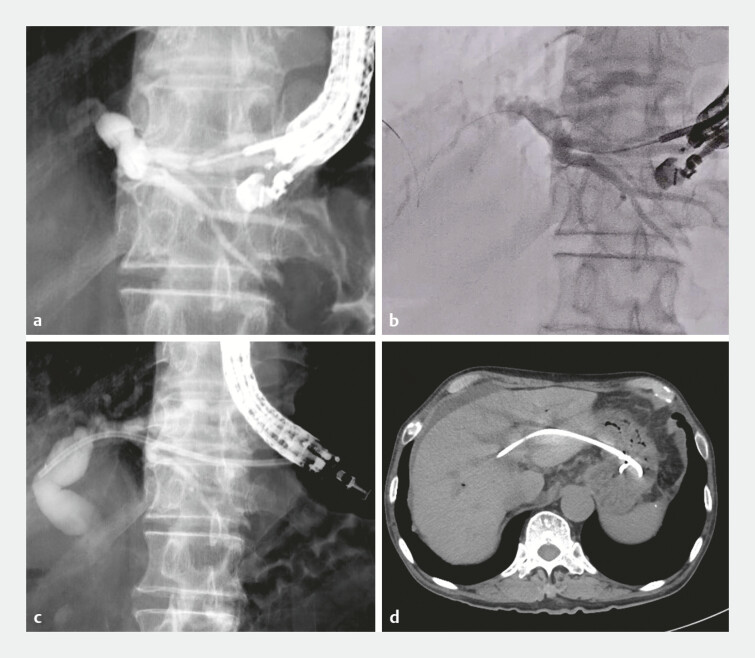
EUS-guided hepaticogastrostomy using the novel guidewire in the second patient. **a** Contrast injection after 19-gauge puncture. **b** Direct insertion of the novel guidewire. **c** A fluoroscopic image showing successful plastic stent placement without any tract dilation. **d** CT scan confirming appropriate plastic stent placement. EUS, endoscopic ultrasound.

This technique was subsequently applied in another patient undergoing EUS-HGS, enabling successful placement without adverse events ( Fig. 3 **a–d** ) and without additional tract dilation.

Overall, the flush surface method enables direct stent deployment without tract dilation using a 0.035-inch guidewire in selected EUS-BD cases. These findings suggest that the guidewire’s outer diameter plays a critical role in facilitating dilation-free stent delivery. Reproducibility and clinical impact should be further investigated.

Endoscopy_UCTN_Code_TTT_1AS_2AH
